# Inflammatory Biomarkers Predicting Contrast-Induced Acute Kidney Injury in Elderly Patients with ST-Segment Elevation Myocardial Infarction

**DOI:** 10.3390/diagnostics15101191

**Published:** 2025-05-08

**Authors:** Suleyman Sezai Yildiz, Gokhan Cetinkal, Erkan Kalendar, Emre Daglioglu, Betul Balaban, Murat Avsar, Omer Sit, Mujdat Aktas, Kadriye Kilickesmez

**Affiliations:** 1 Department of Cardiology, University of Health Science, Prof. Dr. Cemil Tascioglu City Hospital, 34384 Istanbul, Turkey; gokhancetinkal@yahoo.com (G.C.); kalendarerkan@gmail.com (E.K.); emredaglioglu7@gmail.com (E.D.); betulbalaban@yahoo.com (B.B.); muratavsar@yahoo.com (M.A.); dr.omersit@gmail.com (O.S.); mujdat05@gmail.com (M.A.); 2 Department of Cardiology, Ulus Liv Hospital, 34340 Istanbul, Turkey; kadriye11@yahoo.com

**Keywords:** inflammatory biomarkers, contrast-induced acute kidney injury, elderly patients, ST-segment elevation myocardial infarction

## Abstract

**Background:** The inflammatory response is critically important in ST-segment elevation myocardial infarction (STEMI). The systemic immune-inflammation index (SII) and systemic inflammation response index (SIRI), novel inflammatory biomarkers, have been linked to the determination of outcomes in various diseases. The aim of the current study was to examine the relation of the SII and SIRI with contrast-induced acute kidney injury (CI-AKI) in elderly subjects with STEMI undergoing primary percutaneous coronary intervention (pPCI). **Methods:** All patients diagnosed with STEMI between November 2020 and September 2024 were screened, and patients aged over 70 were retrospectively analyzed in the present study. The patients were divided into two groups according to CI-AKI development. The SII and SIRI were calculated based on the peripheral blood counts. A receiver operating characteristic (ROC) curve analysis was performed to determine the sensitivity and specificity of the SII and SIRI in predicting CI-AKI. Additionally, multivariable logistic regression models were employed to investigate the associations between inflammatory indices and the incidence of CI-AKI in elderly patients with STEMI. **Results:** A total of 263 participants were included (mean age 77.67 ± 6.20, 56% women). Both the SII and SIRI were higher in the CI-AKI group than in the non-CI-AKI group (3252 ± 2257, 1097 ± 991 *p* < 0.001 for SII; 12.1 ± 4.54, 4.86 ± 2.42 *p* < 0.006 for SIRI). In the receiver operating characteristic analysis, the SII and SIRI showed the highest area under curve (AUC) compared with other inflammatory parameters. The AUC of the SII and SIRI were 0.903 and 0.867 (*p* < 0.001). In multivariate logistic regression analysis, the SII and SIRI were found as independent predictors of CI-AKI. **Conclusions**: The SII and SIRI were found to be important markers for predicting post-procedural CI-AKI in elderly patients with STEMI.

## 1. Introduction

Contrast-induced acute kidney injury (CI-AKI) is a significant complication that can occur in ST-segment elevation myocardial infarction (STEMI) patients undergoing primary percutaneous coronary intervention (pPCI) [1,2]. The occurrence of CI-AKI has been linked to prolonged hospitalization, the need for renal replacement therapy, major adverse cardiovascular events, and even mortality [1,2,3]. Therefore, predicting CI-AKI using easily calculated markers before the angiographic procedure is essential for identifying high-risk patients and implementing preventive measures [4].

Several risk factors have been associated with CI-AKI development, including female gender, advanced age, congestive heart failure, diabetes mellitus, elevated baseline creatinine levels, anemia, the volume of contrast media used, and the administration of nephrotoxic medications [5,6,7,8,9,10,11]. Several pathways like nephrotoxic effects, inflammatory responses, oxidative stress, reactive oxygen species generation, and renal medullary hypoxia were assumed to play a role in the occurrence of CI-AKI. Consequently, early and precise risk stratification, along with tailored preventive measures, holds significant clinical importance [11].

Recent studies have proposed two new composite indices, the systemic immune-inflammation index (SII) and systemic inflammatory response index (SIRI), which serve as valuable tools for assessing both inflammatory and immune responses simultaneously [12,13]. While these inflammatory markers consistently correlate with unfavorable cancer outcomes, they also show remarkable predictive capacity in cardiovascular conditions. Recent cardiovascular studies particularly highlight their association with poor prognosis, typically measured as composite endpoints. Of clinical significance, the SIRI proved to be a robust, independent prognostic indicator for major adverse clinical events in acute coronary syndrome undergoing pPCI [14,15,16,17].

Currently, no studies have investigated the relation of SIRI and SII with CI-AKI development in elderly STEMI patients undergoing primary PCI. Therefore, we aimed to investigate the relationship between SII, SIRI, and the development of CI-AKI in this specific patient population.

## 2. Materials and Methods

**Figure 1:**
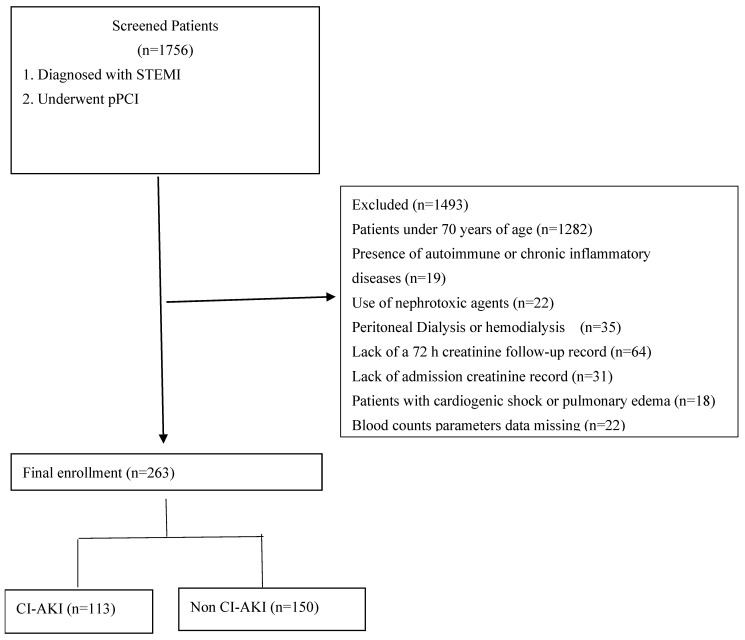
Flow chart of patient enrolment. pPCI, primary percutaneous coronary intervention; CI-AKI, contrast-induced acute kidney injury; STEMI, ST elevation myocardial infarction.

This is a single-center, retrospective, and observational study that consecutively included patients with a history of STEMI who underwent PCI from 3 November 2020 to 1 September 2024. The inclusion criteria were as follows: (1) age ≥ 70 years; (2) patients diagnosed with STEMI, who underwent PCI. The exclusion criteria included patients aged <70 years, presence of autoimmune or chronic inflammatory diseases, use of nephrotoxic agents, peritoneal dialysis or hemodialysis, lack of a 72 h creatinine follow-up record, lack of admission creatinine record, cardiogenic shock or pulmonary edema, and missing laboratory or clinical data. (Figure 1).

The study was conducted following the principles in the Declaration of Helsinki and was approved by the Medical Ethics Committee of the Prof. Dr. Cemil Tascioglu City Hospital of the University of Health Science (approved date/number: 31.12.2024/E-486707771-770-264016808). Informed consent was obtained from all the subjects involved in the study.

In this study, baseline clinical data from the hospital information system were collected for all the patients, including age, sex, and related risk factors (e.g., coronary heart disease, hypertension, diabetes, smoking, atrial fibrillation, and hyperlipidemia). Venous blood samples were collected upon admission to the hospital and during hospitalization. Complete blood count was measured using a Mindray BC6800 plus autoanalyzer (Mindray, Shenzhen, China). Biochemical measurement was performed using a clinical biochemistry analyzer (Beckman AU5800, Beckman Coulter Life Sciences, Brea, CA, USA) in the hospital biochemistry laboratory.

STEMI was defined according to the ESC guidelines for the STEMI criteria and management [18]. The patients underwent coronary angiography with subsequent percutaneous coronary intervention within 90 min of admission to the intensive care unit. Hypertension was defined by a systolic blood pressure ≥140 mmHg and diastolic blood pressure ≥90 mmHg or a history of hypertension or use of antihypertensive drugs [19]. Smoking status was classified as never smoker (defined as smoking less than 100 cigarettes in total), former smoker (defined as smoking more than 100 cigarettes in total and not smoking at all now), and current smoker (defined as smoking more than 100 cigarettes in total and smoking some days or every day). Diabetes was defined by a fasting blood glucose >126 mg/dLol/L or a previous history of diabetes or use of hypoglycemic drugs [20]. Coronary heart disease was defined by a previous history of coronary heart disease. Atrial fibrillation was defined by a previous history of atrial fibrillation or a diagnosis of atrial fibrillation by electrocardiography. Heart failure (CHF) was identified as the presence of characteristic symptoms accompanied by objective signs of cardiac dysfunction, resulting in diminished cardiac output and/or increased intracardiac pressures [21]. Hyperlipidemia was identified as total cholesterol higher than 200 mg/dL and/or low-density lipoprotein cholesterol higher than 130 mg/dL. According to the KDIGO guidelines, CI-AKI is an iatrogenic complication defined as either a ≥0.5 mg/dL absolute or ≥25% relative serum creatinine increase within 48–72 h post-iodinated contrast administration, persisting 2–5 days without alternative causes [22]. The SII was calculated using the following formula: SII = (peripheral platelet counts × neutrophil counts/lymphocyte counts)/1000. The systemic inflammation response index was defined as follows: neutrophil count × monocyte/lymphocyte count [12,23]. These parameters were calculated from complete blood count samples which were delivered from patients on admission at the emergency department of our hospital before the primary PCI. Standard coronary angiography was performed using the Seldinger technique via either the transradial or transfemoral approach according to the operator’s preference. Low-osmolar, non-ionic iohexol (350 mg iodine/mL; 755 mOsm/kg H_2_O; Omnipaque-350, GE Healthcare, Rydalmere, Australia) was utilized as the contrast agent for all the patients. Following the pPCI procedure, in accordance with the hospital protocol to prevent CI-AKI, all the patients received a saline infusion (NaCl 0.9%) as follows: 1 mL/kg/h of normal saline over 12 h for patients without heart failure (HF), and 0.5 mL/kg/h over 12 h for those with HF. The final hydration duration was adjudicated by the treating interventional cardiologist based on individual patient risk assessment. The Bleeding Academic Research Consortium (BARC 3 or 5) criteria were used as a bleeding score.

Statistical analysis was performed using the SPSS 20 software (SPSS Inc., Chicago, IL, USA). The distribution of data data was assessed using the Kolmogorov–Smirnov test. The data for continuous variables were reported as means ± standard deviation. Categorical variables were reported as numbers and percentages. Continuous variables were compared between groups using an independent sample T-test for normally distributed data or Mann–Whitney U-test for abnormally distributed data. Categorical data were compared using the chi-square or Fisher’s Exact test. Univariable logistic regression analysis was used to detect the association of variables with CI-AKI in elderly patients with STEMI. A multivariable logistic regression analysis was performed with clinically relevant variables with *p* < 0.05 in univariable regression analysis. Receiver operating characteristic (ROC) curves were used to compare the performance and predictive accuracy of the SII and SIRI scores for CI-AKI. The optimal test cutoff point was established by calculating Youden’s index. A *p*-value < 0.05 was considered statistically significant.

## 3. Results

This clinical study included a total of 263 elderly patients aged seventy years and above with STEMI who underwent pPCI. Contrast-induced AKI developed in 113 patients (43%) after the pPCI procedure in the elderly patients with STEMI. The mean age of the patient group was 77.5 ± 6.2. Also, the percentage of female patients was 57%. Only 10 patients underwent dialysis. History of smoking, diabetes, and hypertension was 16.7%, 28.5%, and 67.6%, respectively. A total of 98 patients presented with anterior STEMI, while 74 patients had Killip class >1 (Table 1).

Table 2 demonstrates the demographic and clinical characteristics of the CI-AKI (+) and CI-AKI (-) “-” patients. The contrast-induced AKI (+) patients were older than the CI-AKI (-) group (78.7 ± 6.3 vs. 76.6 ± 5.9 years; *p* = 0.005). Likewise, the elderly patients with CI-AKI (+) had higher frequencies of smoking, Killip > 1, CHF, anterior STEMI, bleeding, and vasopressor use compared with CI-AKI (-) “-”. The number of elderly patients with DM, HT, AF, and a previous history of CAD, COPD, peripheral artery disease, and hyperlipidemia was similar between the groups. There was no difference between the groups in terms of the contrast media used and concomitant medications.

Table 3 demonstrates the baseline laboratory measurements of the sample in all the elderly patients.

Table 4 shows laboratory values between groups. Considering biochemical parameters, we could not reach any significant differences between the groups in hemoglobin, glucose at admission, low-density lipoprotein cholesterol, total cholesterol, triglyceride, C-reactive protein at admission, serum creatinine at admission, and monocyte counts. However, the patients developing CI-AKI had higher levels of maximum serum creatinine, maximum CRP, and peak hs troponin I. In addition, the patients with CI-AKI had higher neutrophil and platelet counts and had lower levels of lymphocyte counts compared with those without CI-AKI. Likewise, among the markers calculated from hematological parameters, the SII and SIRI were higher in the patients who developed CI-AKI compared to those who did not (3252.35 ± 225.7 vs. 1097.95 ± 99.1, *p* < 0.001; 12.1 ± 4.54 vs. 2.86 ± 1.48, *p* < 0.006, respectively).

We evaluated the roles of some CI-AKI risk factors using multivariate analysis in the elderly patients with STEMI who underwent pPCI. In a univariate analysis, we found the age, female sex, maximum hsCRP, maximum hsTnI, Killip > 1, glucose at admission, anterior MI, SII, and SIRI to be associated with CI-AKI development. A multivariate logistic regression analysis showed that only SII (OR: 1.008, 95% confidence interval (CI): 1.003–1.020, *p* < 0.001) and SIRI (OR: 1.231, 95% CI: 1.057–1.433, *p* = 0.008) were independent predictors of CI-AKI development (Table 5).

**Figure 2:**
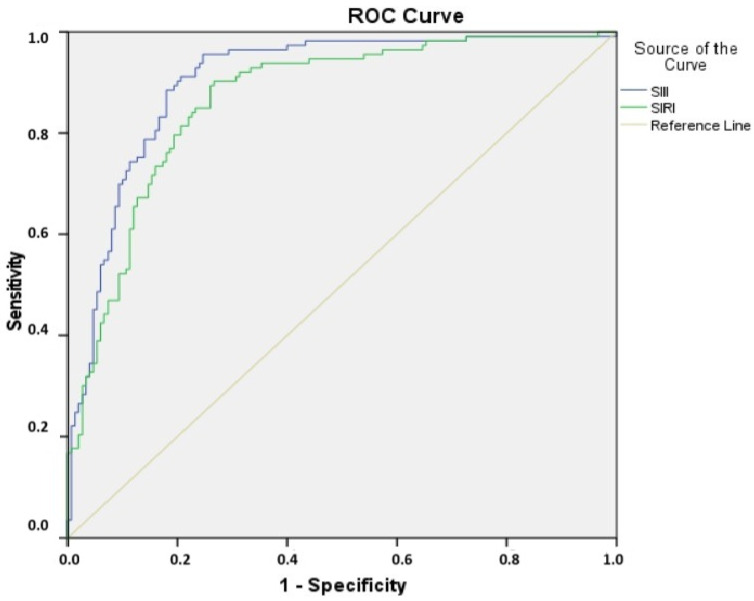
Receiver operating characteristic (ROC) curve with calculated area under the curve and optimal cutoff point for the SII and SIRI to identify the presence of CI-AKI after pPCI in elderly patients with STEMI.

A receiver operating characteristic (ROC) analysis was used to evaluate the predictive level of the SII and SIRI for CI-AKI. The ROC analysis showed that the best cutoff value for the SII to predict the development of CI-AKI, with 79% sensitivity and 84% specificity (area under ROC curve = 0.903 (95% CI: 0.865–0.941), *p* < 0.001), was 1703. The best cutoff value of 3.65 for the SIRI predicted the development of CI-AKI, with a sensitivity of 82% and a specificity of 78% (area under ROC curve = 0.8670 (95% CI: 0.823–0.911), *p* < 0.001) (Figure 2).

## 4. Discussion

In the present study, we analyzed the relationship between the SIRI, SII, and the development of CI-AKI after pPCI in elderly STEMI patients. Our study showed the predictive roles of the systemic immune-inflammation index and systemic inflammation response index, novel inflammatory markers, for contrast-induced acute kidney injury following primary percutaneous coronary intervention in elderly patients with STEMI. In elderly patients with STEMI, our study findings demonstrated that both the SII and SIRI exhibit high sensitivity and specificity in predicting CI-AKI development after pPCI (79% sensitivity and 84% specificity for SII, 82% sensitivity and 78% specificity for SIRI).

Among the STEMI patients who underwent pPCI, the rate of CI-AKI development varied from 15% to 35%, with no significant age-related differences, and the incidence exceeded 50% among high-risk populations, particularly elderly patients and those with comorbid conditions, including diabetes mellitus, heart failure, or chronic kidney disease [24,25,26]. As the global population ages, contrast-enhanced procedures are being increasingly performed in elderly patients worldwide. Contrast-induced acute kidney injury (CI-AKI), a common complication of PCI, represents a leading cause of hospital-acquired renal impairment. The adverse clinical outcomes associated with CI-AKI, including prolonged hospitalization and elevated treatment costs, have significantly constrained the use of contrast angiography, particularly in high-risk STEMI patients [27,28]. While CI-AKI’s pathophysiology is not fully understood, it is associated with nephrotoxicity, systemic inflammation, oxidative stress, reactive oxygen species overproduction, and renal medullary ischemia [28,29]. In addition to the previously mentioned underlying mechanisms in elderly patients, substantial loss of nephrons caused by advanced age, increases in vascular stiffness, and decreases in vascular endothelial function may lead to CI-AKI [30,31,32]. The most commonly used contrast agents for intravascular injection today are either iso-osmolar agents, such as iodixanol (a dimer, approximately 290 mOsm/kg), or low-osmolar nonionic monomers (including iohexol, iomeprol, iopamidol, iopromide, ioversol, and ioxilan), with osmolality ranging from 700 to 850 mOsm/kg. Regarding direct effects, iodine contrast media can induce cytotoxicity in nephrons, including renal tubular epithelial and endothelial cells, leading to mitochondrial dysfunction, apoptosis, pyroptosis, necrosis, and interstitial inflammation. Indirectly, iodine contrast media can alter renal hemodynamics, causing the vasoconstriction of renal vessels and resulting in intramedullary ischemia and hypoxia. This process is exacerbated by massive anti-inflammatory factor release, which induces lymphocyte apoptosis while simultaneously compromising the body’s immune defenses and antioxidant capacity, ultimately resulting in endothelial dysfunction [31,32,33]. Platelet activation contributes significantly to inflammatory processes through the secretion of multiple pro-inflammatory cytokines. Such excessive inflammatory responses may induce systemic microcirculatory disturbances, accelerated platelet consumption, and impaired renal perfusion with consequent hypoxia. These findings collectively suggest that elevated SII and SIRI levels potentially promote CI-AKI pathogenesis through dual mechanisms: sustained inflammatory cascades and dysregulated coagulation pathways [34,35,36,37]. Although the incidence of CI-AKI continues to rise, effective preventive therapies for CI-AKI remain very limited. Currently, intravenous saline solution is the most commonly recommended and used treatment to prevent CI-AKI following pPCI. However, it has been reported that the use of SGLT2 inhibitors significantly reduces the development of CI-AKI, particularly in patients with acute myocardial infarction undergoing pPCI and type 2 diabetes [38,39].

Both the SII and SIRI have been extensively investigated in various cardiovascular diseases [13,40,41,42,43]. Bağcı et al. demonstrated in STEMI patients (mean age 66 years) that the pre-procedurally calculated SII serves as an independent predictor of CI-AKI development following pPCI [41]. In another study, Marci et al. reported a significant association between elevated SII and SIRI values and increased mortality rates in STEMI patients [13]. The literature reveals no established predictive threshold for SII, with studies reporting varying cutoff values. This discrepancy underscores the necessity for standardized large-scale investigations. Notably, Yang et al. demonstrated that elevated SII levels (>694.3 × 10⁹/L) correlate with adverse cardiovascular outcomes post-PCI in CAD patients [43].

Our study demonstrated a 42% incidence of CI-AKI following pPCI in STEMI patients aged over 70 years. The patients who developed CI-AKI had significantly higher comorbidity burden compared to those without CI-AKI. In STEMI, as a manifestation of acute coronary syndrome, inflammatory processes secondary to coronary plaque rupture play a fundamental role. Neutrophils, platelets, monocytes, and lymphocytes are known to be key mediators in this process, all of which can be easily and inexpensively measured via a complete blood count [8,13,15,21,30,33,41,42]. In our patient cohort, the neutrophil and platelet counts were higher in the group that developed CI-AKI compared to those who did not, whereas the lymphocyte counts were lower. The monocyte counts showed no significant difference between the groups. Both the systemic immune-inflammation index (SII) and the systemic inflammatory response index (SIRI), calculated from neutrophil, platelet, monocyte, and lymphocyte values, were significantly elevated in the CI-AKI group. To our knowledge, this is the first study to demonstrate that both the SII and SIRI independently predict the development of CI-AKI following pPCI in elderly patients with STEMI. Based on our findings, we speculate that calculating pre-procedural SII and SIRI values in elderly patients diagnosed with STEMI may allow for the early identification of those at high risk for CI-AKI after pPCI. This, in turn, could facilitate timely interventions, potentially shortening hospital stays and even contributing to a reduction in mortality. The SII and SIRI may offer a more comprehensive and reliable reflection of the immune system’s overall status in cardiovascular disease compared to NLR and PLR [13]. In this study, we employed a combined index approach and demonstrated that both the SII and SIRI correlate with the development of CI-AKI after pPCI in elderly STEMI patients. Notably, the SII and SIRI emerged as stronger predictors of CI-AKI in elderly STEMI patients. Specifically, when the SII and SIRI values exceeded 1703 and 3.65, respectively, the risk of CI-AKI after pPCI in elderly STEMI patients showed a statistically significant increase. This study has some limitations. First of all, the retrospective and single-center nature of the study leads to both selection bias and generalizability problems. Prospective data collection in such a patient population could have provided more reliable results. In addition, the very limited sample size may reduce the stability and power of the model, especially in multivariate regression analyses. Secondly, patients were not stratified by age or any clinical risk scores for further analysis. Despite these limitations, our study has the strength that the SII and SIRI, which were known as novel and robust inflammatory markers, predict CI-AKI development after PCI in elderly STEMI patients while laying the groundwork for future research to validate and expand upon these findings.

In conclusion, the SII and SIRI have a certain predictive value in evaluating the occurrence of CI-AKI after pPCI in elderly patients with STEMI. This method has the advantages of convenient detection and low cost and has a good application prospect in predicting CI-AKI after PCI in elderly patients with STEMI.

## Figures and Tables

**Table 1:** The demographic and clinical features of elderly patients with STEMI.

	All
	(*n* = 263)
Variables	*n* (%)
Female	150 (57%)
Age(years)	77.5 ± 6.2
Smoking	44 (16.7%)
Diabetes	75 (28.5%)
Dyslipidemia	52 (19.8%)
Hypertension	178 (67.7%)
AF	28 (10.6%)
Killip class ˃ 1	74 (28.1%)
Previous history of CAD	92 (34.9%)
COPD	33 (12.6%)
Periferal artery disease	12 (4.6%)
Malignancy	20 (7.6%)
CHF	25 (9.5%)
Anterior STEMI	98 (37.2%)
Bleeding	11 (4.2%)
Vasopressor and inotrope Use	59 (22.4%)
Contrast media (ml)	166.7 ± 41.8
ACEI/ARB	90 (34.2%)
Beta blockers	145 (55.1%)
Statins	215 (81.7%)
Ticagrelor	107 (40.7%)
Clopidogrel	156 (59.3%)

**Abbreviations:** ACEI, angiotensin-converting enzyme inhibitor; ARB, angiotensin receptor blocker; CAD, coronary artery disease; CHF, congestive heart failure; CI-AKI, contrast-induced acute kidney injury; COPD, chronic obstructive pulmonary disease. Killip classification (risk assessment of patients after STEMI), Killip class I: patients without any clinical sign of heart failure; Killip class II: patients with crackles or rales in the lungs, elevated jugular venous pressure, and an S3 gallop; Killip class III: patients with evident acute pulmonary edema; Killip class IV: patients with cardiogenic shock or hypotension (systolic blood pressure < 90 mmHg) and features of low cardiac output. STEMI, ST elevation myocardial infarction.

**Table 2:** The demographic and clinical features of patients with and without CI-AKI in elderly patients with STEMI.

	CI-AKI (*n* = 113)	Non-CI-AKI (*n* = 150)	*p* Value
Variables *n* (%)			
Female	64 (56.6%)	86 (57.3%)	0.726
Age (years)	78.7 ± 6.3	76.6 ± 5.9	0.005
Smoking	32 (21%)	12 (11%)	0.02
Diabetes	34 (30.1%)	41 (27.3%)	0.624
Dyslipidemia	27 (24%)	25 (17%)	0.145
Hypertension	96 (64%)	82 (73%)	0.141
AF	12 (10.6%)	16 (10.7%)	0.975
Killip class ˃ 1	41 (36.3%)	33 (22%)	0.011
Previous history of CAD	42 (38.7%)	50 (33%)	0.149
COPD	12 (10.6%)	21 (14%)	0.413
Periferal artery disease	6 (5.3%)	6 (4%)	0.614
Malignancy	9 (8%)	11 (7%)	0.853
CHF	16 (14.2%)	9 (6%)	0.026
Anterior STEMI	50 (44%)	48 (32%)	0.046
Bleeding	8 (7.1%)	3 (2%)	0.042
Vasopressor and inotrope Use	34 (30%)	25 (17%)	0.01
Contrast media (ml)	167.8 ± 38.4	163.3 ± 43.6	0.667
ACEI/ARB	40 (35.4%)	50 (33%)	0.223
Beta blockers	62 (54.9%)	83 (55.3%)	0.824
Statins	90 (79.6%)	125 (83.3%)	0.882
Ticagrelor	45 (39.8%)	62 (41.3%)	0.726
Clopidogrel	68 (60.2%)	88 (58.7%)	0.741

**Abbreviations:** ACEI, angiotensin-converting enzyme inhibitör; ARB, angiotensin receptor blocker; CAD, coronary artery disease; CHF, congestive heart failure; CI-AKI, contrast-induced acute kidney injury; COPD, chronic obstructive pulmonary disease. Killip classification (risk assessment of patients after STEMI), Killip class I: patients without any clinical sign of heart failure; Killip class II: patients with crackles or rales in the lungs, elevated jugular venous pressure, and an S3 gallop; Killip class III: patients with evident acute pulmonary edema; Killip class IV: patients with cardiogenic shock or hypotension (systolic blood pressure < 90 mmHg) and features of low cardiac output. STEMI, ST elevation myocardial infarction.

**Table 3:** The laboratory features of elderly patients with STEMI.

Laboratory Data	All (*n* = 263)
White blood cell count, ×10^3^/μL	11.93 ± 4.24
Hemoglobin, g/dL	12.71 ± 2.1
Platelet count, ×10^3^/μL	260 ± 30.38
Neutrophil count, ×10^3^/μL	8.99 ± 3.78
Lymphocyte count, ×10^3^/μL	1.76 ± 1.19
Monocyte count, ×10^3^/μL	1.23 ± 0.84
Serum creatinine at admission, mg/dL	1.31 ± 0.83
Maximum serum creatinine, mg/dL	1.76 ± 1.26
CRP at admission, mg/L	25.9 ± 6.3
Maximum CRP, mg/L	70.1 ± 58.6
Glucose at admission, mg/dL	125.8 ± 49.8
Troponin I at admission	2527 ± 1074
Peak troponin I	7553 ± 4933
LVEF	46.7 ± 10.3
SII	2023.61 ± 196.92
SIRI	6.83 ± 2.69

**Abbreviations:** CI-AKI, contrast-induced acute kidney injury; CRP, C-reactive protein; SII, systemic immune-inflammation index; SIRI, systemic inflammation response index; LVEF, left ventricular ejection fraction.

**Table 4:** The laboratory features of patients with and without CI-AKI in elderly patients with STEMI.

	CI-AKI	Non-CI-AKI	*p* Value
	(*n* = 113)	(*n* = 150)	
Laboratory Data			
White blood cell count, ×10^3^/μL	13.22 ± 4.52	10.4 ± 3.26	<0.001
Hemoglobin, g/dL	12.49 ± 2.1	12.85 ± 2.01	0.174
Platelet count, ×10^3^/μL	286.93 ± 81.8	239.65 ± 73	<0.001
Neutrophil count, ×10^3^/μL	10.81 ± 4.14	7.62 ± 2.8	<0.001
Lymphocyte count, ×10^3^/μL	1.07 ± 0.38	2.27 ± 1.0	<0.001
Monocyte count, ×10^3^/μL	1.34 ± 1.0	1.17 ± 0.82	0.134
Serum creatinine at admission, mg/dL	1.29 ± 0.58	1.32 ± 0.68	0.765
Maximum serum creatinine, mg/dL	2.23 ± 1.32	1.39 ± 1.1	<0.001
CRP at admission, mg/L	25.53 ± 6.8	26.29 ± 5.0	0.905
Maximum CRP, mg/L	90.37 ± 69.3	66.94 ± 50	<0.001
Glucose at admission, mg/dL	125.5 ± 48.9	126.5 ± 50.1	0.892
Troponin I at admission	2673 ± 1123	2420 ± 1038	0.790
Peak troponin I	10100 ± 6827	4529 ± 3562	0.003
LVEF	44.10 ± 10.8	48.60 ± 9.80	<0.003
SII	3252.35 ± 225.7	1097.95 ± 99.1	<0.001
SIRI	12.1 ± 4.54	2.86 ± 1.48	<0.006

**Abbreviations:** CI-AKI, contrast-induced acute kidney injury; CRP, C-reactive protein; SII, systemic immune-inflammation index; SIRI, systemic inflammation response index; LVEF, left ventricular ejection fraction.

**Table 5:** Univariate and multivariate logistic regression analyses showing the independent predictors of CI-AKI after pPCI in elderly patients with STEMI.

	Univariate		Multivariate	
	OR (95%CI)	*p* Value	OR (95%CI)	*p* Value
Age	1.058 (1.016–1.101)	0.006	1.030 (0.967–1.097)	0.356
Gender (female)	1.627 (1.008–2.699)	0.05		
Max. CRP	1.006 (1.003–1.009)	<0.001	1.004 (0.998–1.010)	0.222
Max. hsTnI	1.006 (1.002–1.010)	0.003	1.005 (0.997–1.016)	0.227
Hemoglobin	0.922 (0.819–1.037)	0.175		
Killip > 1	2.019 (1.171–3.480)	0.011	0.940 (0.355–2.488)	0.901
Glucose at admission	1.005 (1.001–1.010)	0.022	1.002 (0.996–1.008)	0.462
Creatinine at admission	0.955 (0.707–10290)	0.765		
Anterior STEMI	1.670 (1.007–2.769)	0.047	1.282 (0.564–2.916)	0.553
SII	1.007 (1.001–1.011)	<0.001	1.008 (1.003–1.020)	<0.001
SIRI	1.569 (1.401–1.757)	<0.001	1.231 (1.057–1.433)	0.008

**Abbreviations:** Max-CRP, maximum high-sensitivity C-reactive protein; Max hsTnI, maximum high-sensitivity troponin I; OR, odds ratio; SII, systemic immune-inflammation index; SIRI, systemic inflammation response index.

## Data Availability

The data sets used and/or analyzed in the present study are available from the corresponding author upon request.

## Abbreviations

The following abbreviations are used in this manuscript:

SII	systemic immune-inflammation index
SIRI	systemic inflammation response index
CI-AKI	contrast-induced acute kidney injury
STEMI	ST-segment elevation myocardial
pPCI	primary percutaneous coronary intervention
